# Probiotics for prevention and treatment of respiratory tract infections in children: A systematic review and meta-analysis of randomized controlled trials

**DOI:** 10.1097/MD.0000000000004509

**Published:** 2016-08-07

**Authors:** Yizhong Wang, Xiaolu Li, Ting Ge, Yongmei Xiao, Yang Liao, Yun Cui, Yucai Zhang, Wenzhe Ho, Guangjun Yu, Ting Zhang

**Affiliations:** a Department of Gastroenterology, Hepatology, and Nutrition; b Pediatric Intensive Care Unit, Shanghai Children's Hospital, Shanghai Jiao Tong University, Shanghai, PR China; c Department of Pathology and Laboratory Medicine, Temple University School of Medicine, Philadelphia, PA; d Department of Children's Healthcare, Shanghai Children's Hospital, Shanghai Jiao Tong University, Shanghai, PR China.

**Keywords:** children, probiotics, randomized controlled trials, respiratory tract infections

## Abstract

**Background:**

Respiratory tract infections (RTIs) represent one of the main health problems in children. Probiotics are viable bacteria that colonize the intestine and affect the host intestinal microbial balance. Accumulating evidence suggests that probiotic consumption may decrease the incidence of or modify RTIs. The authors systematically reviewed data from randomized controlled trials (RCTs) to investigate the effect of probiotic consumption on RTIs in children.

**Methods:**

MEDLINE/PubMed, Embase, Cochrane Library, and Web of Science were systematically searched for RCTs regarding the effect of probiotics on RTIs in children. The outcomes included number of children experienced with at least 1 RTI episode, duration of illness episodes, days of illness per subject, and school/day care absenteeism due to infection. A random-effects model was used to calculate pooled relative risks, or mean difference (MD) with the corresponding 95% confidence interval (CI).

**Results:**

A total of 23 trials involving 6269 children were eligible for inclusion in the systematic review. None of the trials showed a high risk of bias. The quality of the evidence of outcomes was moderate. The age range of subjects was from newborn to 18 years. The results of meta-analysis showed that probiotic consumption significantly decreased the number of subjects having at least 1 RTI episode (17 RCTs, 4513 children, relative risk 0.89, 95% CI 0.82–0.96, *P* = 0.004). Children supplemented with probiotics had fewer numbers of days of RTIs per person compared with children who had taken a placebo (6 RCTs, 2067 children, MD −0.16, 95% CI −0.29 to 0.02, *P* = 0.03), and had fewer numbers of days absent from day care/school (8 RCTs, 1499 children, MD −0.94, 95% CI −1.72 to −0.15, *P* = 0.02). However, there was no statistically significant difference of illness episode duration between probiotic intervention group and placebo group (9 RCTs, 2817 children, MD −0.60, 95% CI −1.49 to 0.30, *P* = 0.19).

**Conclusion:**

Based on the available data and taking into account the safety profile of RCTs, probiotic consumption appears to be a feasible way to decrease the incidence of RTIs in children.

## Abstract 2

Supplemental Digital Content is available in the text

## 1 Introduction

Respiratory tract infections (RTIs) remain one of the leading causes of global morbidity and mortality among children at different ages. Most children younger than 2 years experience several RTIs during the first year of life, and one-quarter suffer from recurrent or prolonged infections in developed countries.^[1,2]^ RTIs are a major cause for parental concern and medical visits in preschool and elementary school children, leading to school absenteeism and hospitalizations.^[3,4]^ They also lead to inappropriate prescription of antibiotics in pediatric practice because antibiotics are not effective against viruses.^[5,6]^ Inappropriate and wide use of antibiotics may lead to the development of bacterial resistance and disturb the normal balance of human microbiota, facilitating the pathogen colonization and reducing availability of vaccines for viruses.^[7,8]^ Economic impact of RTIs is also significant among countries.^[9,10]^ Therefore, RTIs in children are still an important global challenge for public health.

Probiotics are defined by the World Health Organization as live microorganisms that, when administered in adequate amounts, confer a health benefit on the host.^[11]^ The most commonly used probiotics are *Lactobacillus* and *Bifidobacterium* species, followed by the genera *Streptococcus*, *Enterococcus*, *Propionibacterium*, *Bacillus*, and *Escherichia coli*.^[12]^ In addition, some yeast species are used as probiotics, for example, *Saccharomyces boulardii* and *Saccharomyces cerevisiae* are frequently used to treat gastrointestinal disorders.^[13,14]^ A well-characterized probiotic should be defined clearly by the genus, species, and strain designation, as well as indicate the microbiological culture conditions.^[14]^ Probiotic products may be formulated as capsules, tablets, powders (which are regulated as a dietary supplement), and a food ingredient (e.g., yogurts, kefirs, or a drug).^[15]^

Probiotics may exert a wide range of beneficial effects, such as balancing the host gut microbiota and interacting with the innate and adaptive immune system, which may promote resistance against pathogens.^[16]^ In the past few years, probiotics have been widely used in health conditions of RTIs, gastrointestinal, and urogenital tract infections, allergies, necrotizing enterocolitis in preterm infants, infantile colic, autoimmune diseases, and irritable bowel syndrome (IBS).^[13,17–22]^ A lot of clinical studies focusing on evaluating the health benefits of probiotic foods containing well-defined probiotic strains have been conducted in many countries. For example, *S boulardii*, *Lactobacillus rhamnosus* GG, and *Lactobacillus reuteri* DSM 17938 were used to treat acute gastroenteritis, IBS, and antibiotic-associated diarrhea in children and adult patients.^[23–26]^*Bifidobacterium animalis* subsp. *lactis* strain BB-12 was used to prevent nosocomial infections, and *Bifidobacterium lactis* DN-173 010 was used to treat functional constipation in children.^[27]^ The effects of probiotic products may depend on the amount ingested and the pattern of consumption. So far, studies evaluating the effect of probiotics on RTI infection suggested that probiotic consumption may decrease the incidence of RTIs in children. However, conflicting results existed among these studies. In order to provide the latest and convincing evidence, we systematically reviewed the current available data from randomized controlled trials (RCTs) to investigate the effect of probiotic consumption on RTIs in children.

## 2 Methods

The present systematic review and meta-analysis was conducted and reported in adherence to the Preferred Reporting Items for Systematic Reviews and Meta-Analyses statement,^[28]^ and the guidelines of the *Cochrane Handbook for Systematic Reviews of Interventions*.^[29]^ As current study was a review of published studies, ethical approval or patient consent was not necessary.

### 2.1 Search criteria and study identification

Electronic databases MEDLINE/PubMed, Embase, Cochrane Library, and Web of Science were searched for records that compared probiotics to placebo in RTIs in children with key words *probiotic* or *probiotics*, and *respiratory tract infections* or *respiratory infections*, and *children* or *child*. The databases were screened for publications from the earliest available date to April 30, 2016. We also manually checked the cited references of retrieved studies and previous reviews to identify any additional eligible studies. Studies eligible for inclusion were RCTs of any duration comparing probiotic strains, single or combined, consumed by any form of administration, with placebo in apparently healthy children (from birth to 18 years), who developed upper respiratory tract infections (URTIs) or lower respiratory tract infections (LRTIs) at some point during the study. Open or blind trials were eligible, provided that patients were randomized. Probiotic products administered at any dose, single or mix strains, were eligible. Probiotics combined with functional ingredients other than prebiotics (e.g., vitamins) were excluded. To be eligible for inclusion, studies had to be published in English language and the results had to show ≥1 study objectives. Exclusion criteria were clinical trials with adults, animal studies, studies in children who had acquired or congenital immune deficiency, or chronic illness, publications such as comments, editorials, or letters, studies with results from affected organs other than the respiratory tract, duplicated studies, annals of congresses, inappropriate study designs (observational studies, nonrandomized trials), and studies published in languages other than English. Each identified article was initially analyzed by title and abstract, and the eligible articles were selected for full reading. The detailed search strategies are provided in supplementary file (Supplemental Content 1, Fig. S1).

### 2.2 Date extraction and quality assessment

Two of the authors (YW and XL) independently extracted relevant data from each included trial by using predesigned data collection forms on Microsoft Excel, which was confirmed by the third author (TG). These included baseline and demographic data such as author, publication year, study setting site, age, sex, study population, total number of subjects randomized and included in the analysis, and outcomes of interest. Discrepancies between authors were resolved by consensus. The Cochrane Risk-of-Bias Tool was used to assess the risk of bias of each RCT.^[30]^ Publication bias was assessed by visually inspecting funnel plot in each meta-analysis, and by using Egger test when >10 studies were included in a meta-analysis. The quality of the evidence of outcomes was rated by Grading of Recommendations Assessment, Development, and Evaluation (GRADE) approach.^[31]^

### 2.3 Statistical analysis

To evaluate the effect of probiotics in the number of subjects with at least 1 RTI episode, relative risks were calculated for the incidence of RTI between intervention and placebo groups. Means and standard deviations (SDs) were collected for continuous outcomes (duration of illness episodes, number of days of illness, and number of days absent). For studies that did not show the SD data, a SD was imputed using *P* values or SD from other published reviews. For outcomes that showed as means with ranges, SD was calculated from the *P* values, the sample size, median, range and/or interquartile range, or confidence intervals (CIs).^[29,32]^ For studies involving 2 probiotic treatment arms, the means and SDs from the 2 groups were combined to create a single pairwise comparison with the placebo group.^[32]^ Where appropriate, data from all the studies were pooled in a meta-analysis to determine the overall effect size (weighted mean difference [MD]) with 95% CI using a random-effects model. A *P* value <0.05 was considered as statistically significant, except where otherwise specified. Heterogeneity across the studies was investigated using the χ^2^ test (significance set at *P* < 0.05) and the I^2^ statistic (with a value of >50%), and by examining the random-effects between-study variance (t^2^). All the statistical analyses were performed using the Stata 12.0 (Stata Corporation, College Station, TX), GRADE profiler 3.6.1 (GRADE Working Group), and RevMan 5.3 software (The Nordic Cochrane Centre, The Cochrane Collaboration, Copenhagen, Denmark).

## 3 Results

### 3.1 Characteristics of included studies

**Figure 1:**
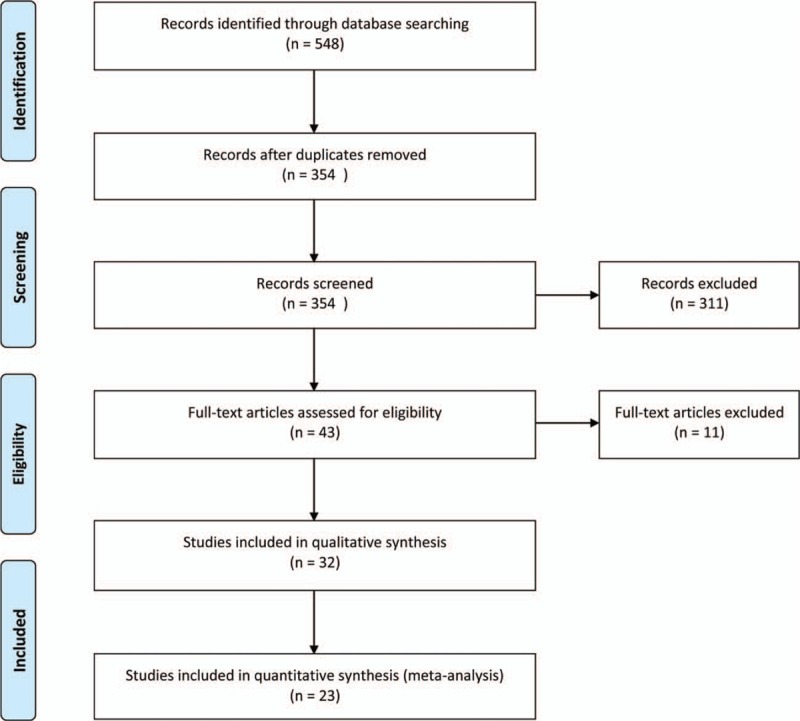
Selection process for the studies included in the meta-analysis.

As shown in Fig. 1, a total of 548 records were identified in the 4 major electronic databases (MEDLINE/PubMed, Embase, Cochrane Library, and Web of Science); 43 studies were considered to be potentially relevant after assessment based on the title and abstract. Inclusion criteria were met by 23 RCTs^[33–55]^ after analyzing full text for eligibility, which were used to identify the intervention and outcomes of this systematic review. The main characteristics of included studies are described in Table 1  . Included studies were published between 2001 and 2016. All trials were randomized, double-blinded, and placebo-controlled. Eighteen trials^[34–44,46,48,50–54]^ were conducted in Europe, 3 in Asia,^[45,49,55]^ 1 in North America,^[47]^ and 1 in South America.^[33]^ Of these trials, 8 trials^[33,35,36,38,44,50,51,54]^ investigated the effect of probiotics on RTIs, including URTIs and LRTIs, and 15 trials^[34,37,39–43,45–49,52,53,55]^ investigated the effect of probiotics on common infections in children, including common winter diseases, cold and influenza-like symptoms, RTIs, and gastrointestinal infections. The duration of probiotic treatment ranged from 5 days to 12 months, and most trials were carried out for >3 months during the winter months. Seven trials^[33,37,39,40,44,51,55]^ used *Lactobacillus* strains, 5 trials^[41,42,52,53,55]^ used *Bifidobacterium* strains, 1 trial^[46]^ used *Lactobacillus fermentum* strain, and 11 trials ^[34–36,38,43,45,47–50,54]^ used a mixture of probiotic strains. Three studies^[45,51,55]^ used separate arms with different probiotic strains compared with 1 placebo group. The included trials evaluated “common cold,” “acute respiratory infections,” “acute otitis media” (AOM), “acute infectious diseases,” and “gastrointestinal tract and respiratory infections.” The descriptions of the symptoms and diagnoses of these conditions were reported clearly by authors of all studies. Study quality assessment of trials by the Cochrane Risk-of-Bias Tool is summarized in Table 2. All 23 trials were described as double blind and provided the blinding methods. Appropriate randomization methods were used in all trials, such as a randomization list generated by computer or by a random number. Appropriate allocation concealment was reported by most studies, including the use of sealed envelopes, and/or use of encoded containers/packages that were identical in appearance except 4 trials^[37,42–44]^ with color coding of study product and placebo. Furthermore, an intention-to-treat analysis was included in most trials. Overall, most trials were considered to have a low risk of bias, and no trials showed a high risk of bias (Table 3). There was no evidence of significant publication bias as funnel plots were roughly symmetrical (Figs. S2–S5). And Egger test was performed for the meta-analysis of the outcome of number of subjects having at least 1 RTI episode (*P* = 0.467). The quality of the GRADE evidence for outcomes of number of subjects with at least 1 illness episode, duration of illness episodes, and number of days absent was moderate, and for number of days of illness was high (Table S1).

**Table 1:**
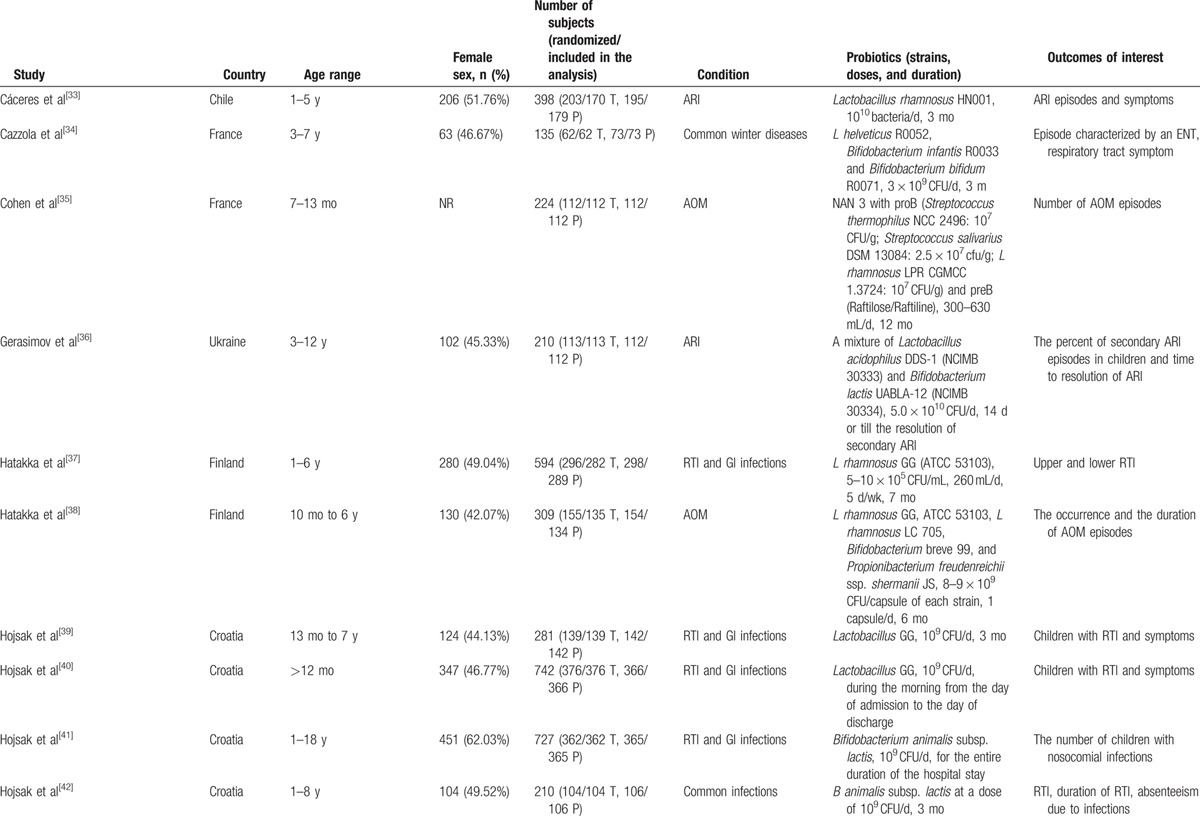
Characteristics of the included studies.

**Table 1 (Continued):**
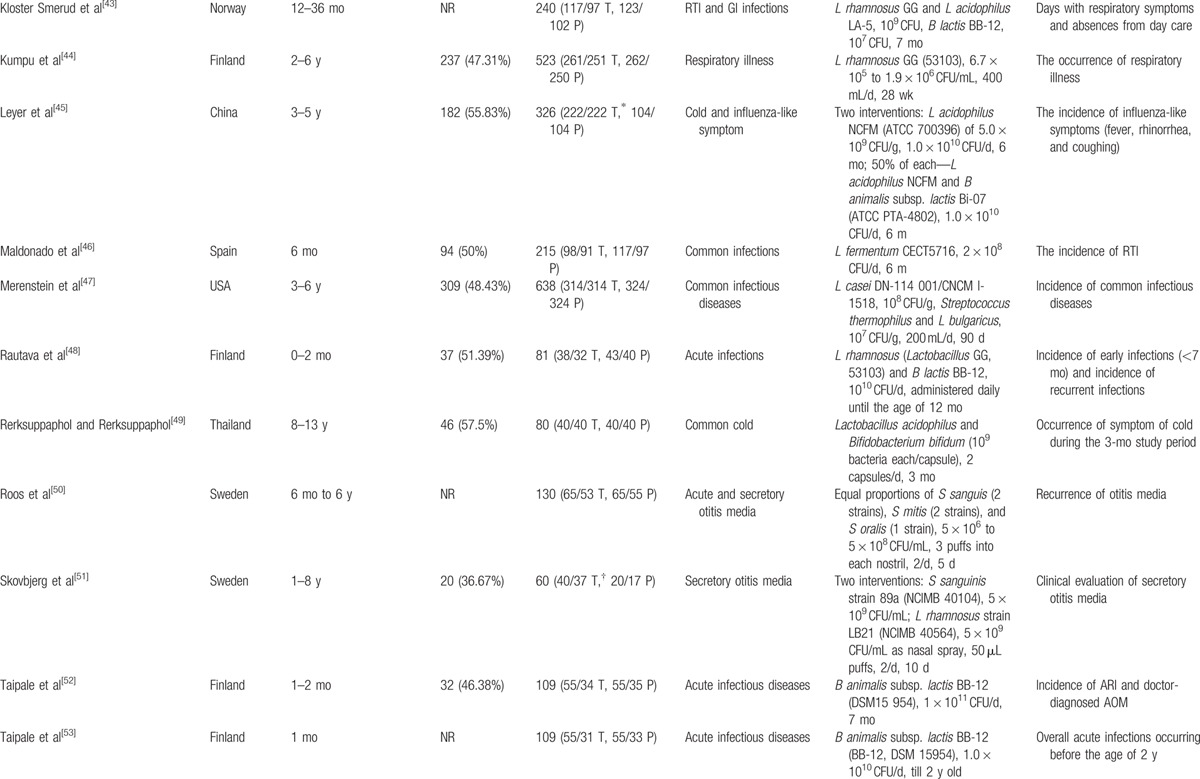
Characteristics of the included studies.

**Table 1 (Continued):**
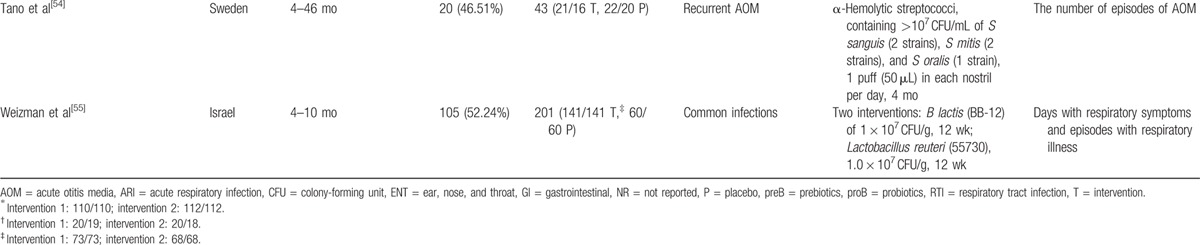
Characteristics of the included studies.

**Table 2:**
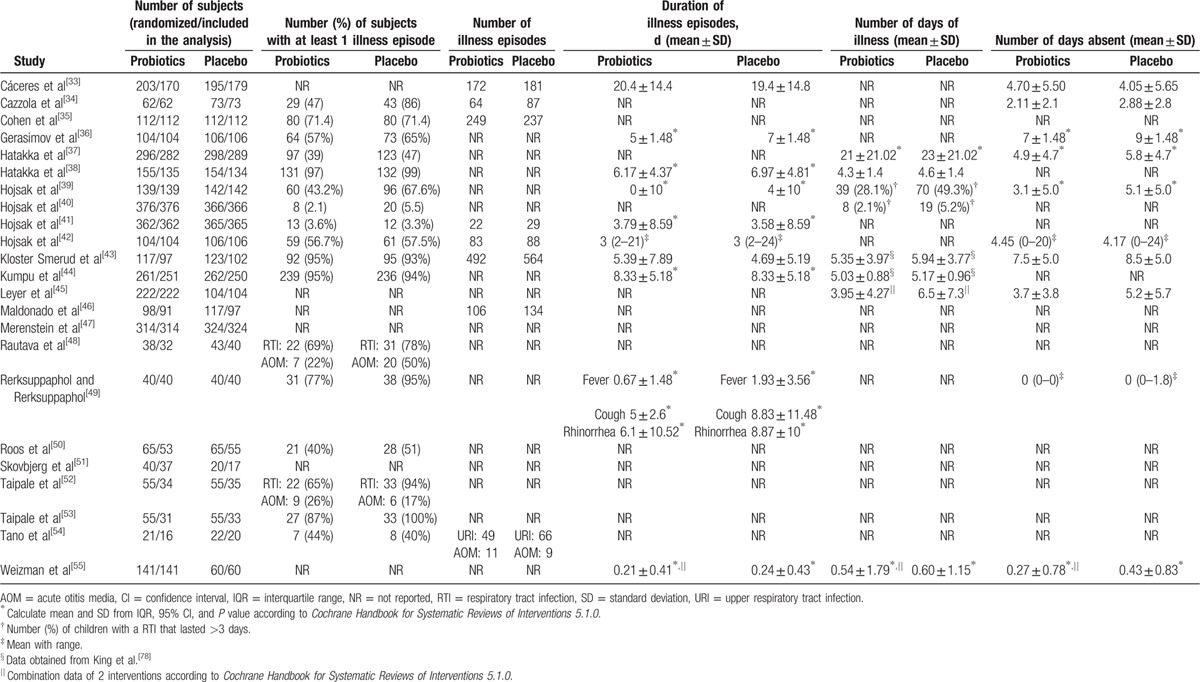
Outcome data of included studies.

**Table 3:**
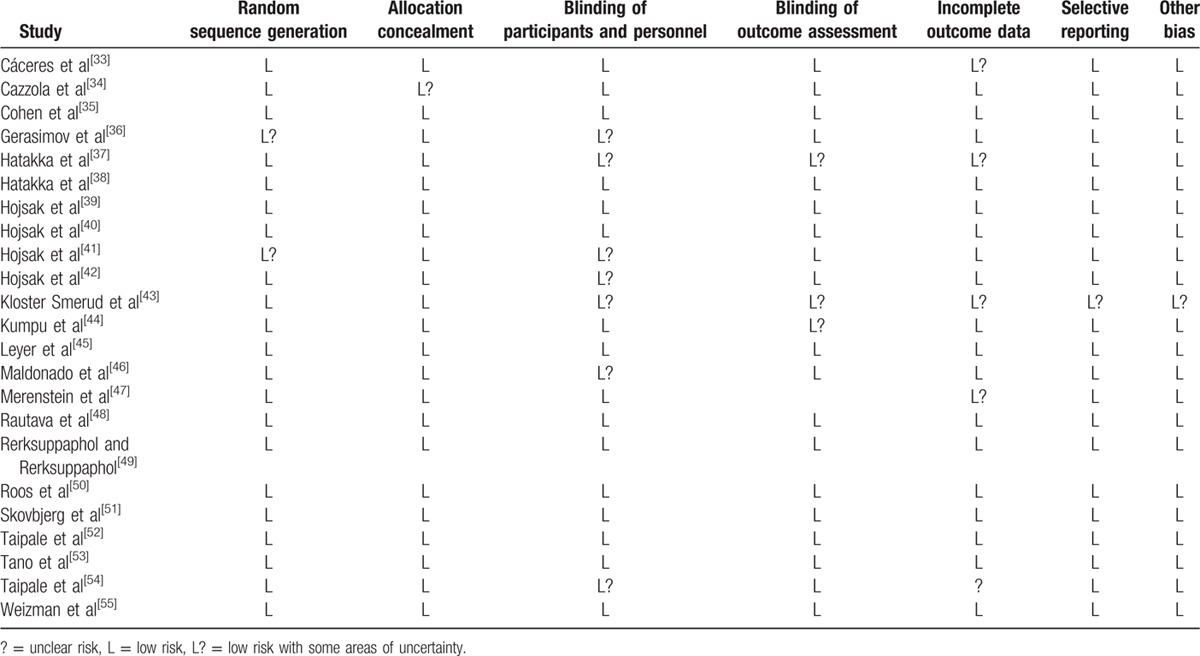
Risk of bias assessment of the included studies.

### 3.2 Effect of probiotics on the number of subjects having at least 1 RTI episode

**Figure 2:**
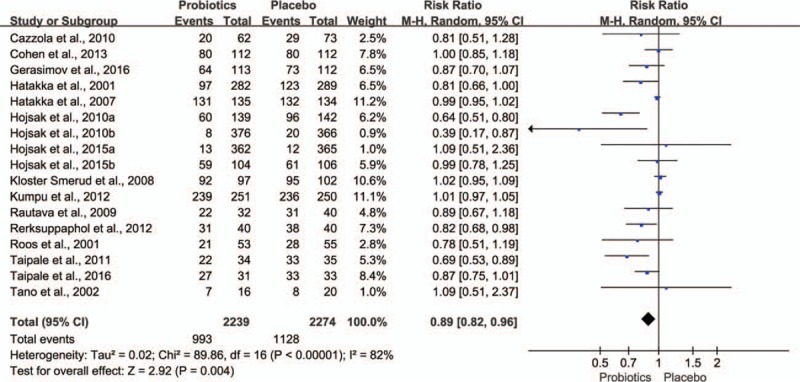
Effect of probiotics on the number of subjects who had at least 1 RTI episode. The “total” is the number of subjects included in the analysis in probiotics and placebo group. CI = confidence interval, M-H = mantel-haenszel, RTI = respiratory tract infection.

Seventeen trials^[34–44,48–50,52–54]^ including 4513 children reported that the number of subjects had at least 1 respiratory symptom episode during the study period. As shown in Fig. 2, probiotic supplementation had a significant effect on the reduction of number of subjects having at least 1 respiratory symptom episode (relative risk 0.89, 95% CI 0.82–0.96, *P* = 0.004). However, there was a statistical heterogeneity among these trials (τ^2^ = 0.02, *P* < 0.00001, I^2^ = 82%).

### 3.3 Effect of probiotic on the duration of RTI illness episodes

**Figure 3:**
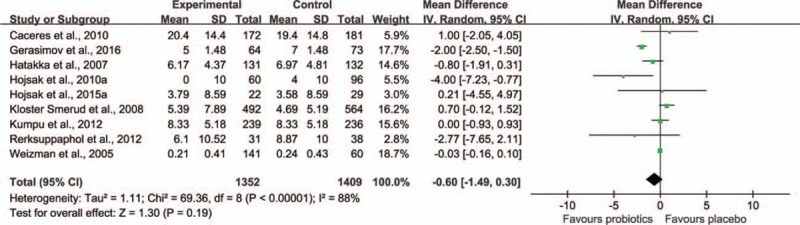
Effect of probiotic on the duration of RTI illness episodes. The “total” is the overall number of illness episodes experienced by the participants,^[33,41,43]^ the number of participants with at least 1 illness episode,^[36,38,39,44,49]^ or the number of participants included in the analysis.^[55]^ CI = confidence interval, RTI = respiratory tract infection, SD = standard deviation.

Among the included trials, 9 trials^[33,36,38,39,41,43,44,49,55]^ including 2817 children reported the data on the duration of illness episode, which was defined as the total sum of illness episode duration (in days) divided by the total number of illness episodes experienced by the study subjects. The data from this 9 trials were pooled to test for overall effect; the results showed that there was no significant statistical difference of illness episode duration between probiotic intervention group and placebo group (weighted MD −0.60, 95% CI −1.49 to 0.30, *P* = 0.19; Fig. 3). Also there was a statistical heterogeneity among these trials (τ^2^ = 1.11, *P* < 0.00001, I^2^ = 88%).

### 3.4 Effect of probiotic on the number of days of RTI illness

**Figure 4:**
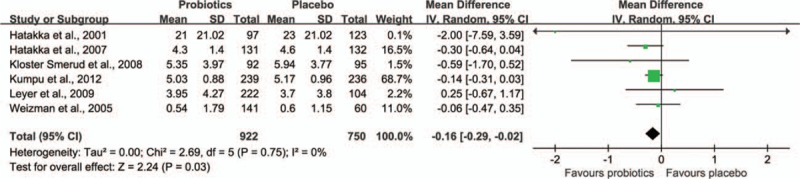
Effect of probiotic on the number of days of RTI illness. The “total” is the number of participants with at least 1 illness episode,^[37,38,43]^ or the number of participants included in the analysis.^[45,55]^ CI = confidence interval, RTI = respiratory tract infection, SD = standard deviation.

Six trials^[37,38,43–45,55]^ including 2067 children reported on the number of days the children were ill. As shown in Fig. 4, the meta-analysis revealed a significant difference in favor of probiotics prevention group. Children supplemented with probiotics had fewer number of days of RTIs per person compared with children who had taken a placebo (weighted MD −0.16, 95% CI −0.29 to 0.02, *P* = 0.03). There was no statistical heterogeneity between the included studies (τ^2^ = 0.00, *P* = 0.75, I^2^ = 0%).

### 3.5 Effect of probiotic on the days absent from day care/school

**Figure 5:**
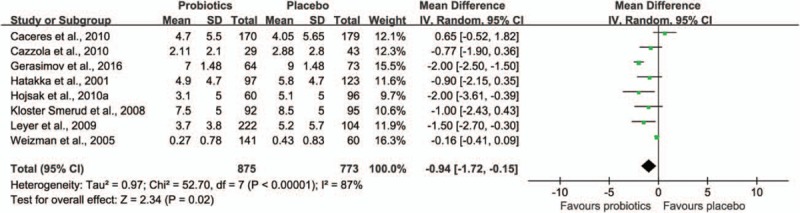
Effect of probiotic on the days absent from day care/school. The “total” is the number of participants with at least 1 illness episode,^[34,36,37,39,43]^ or the number of participants included in the analysis.^[33,45,55]^ CI = confidence interval, SD = standard deviation.

Eight trials^[33,34,36,37,39,43,45,55]^ including 1499 children reported on the number of days absent from day care/school. As shown in Fig. 5, the meta-analysis revealed that children supplemented with probiotics had fewer numbers of days absent from day care/school compared with children who had taken a placebo (weighted MD −0.94, 95% CI −1.72 to −0.15, *P* = 0.02). There was a statistical heterogeneity between the included studies (τ^2^ = 0.97, *P* < 0.00001, I^2^ = 87%).

### 3.6 Adverse events

There were 3 trials^[33,38,43]^ with no adverse events reported. Eleven trials^[36,37,39–42,45,46,49,51,55]^ did not identify any adverse events related to study product including probiotic and placebo during the study period. Nine trials^[34,35,44,47,48,50,52–54]^ showed mild adverse events, such as diarrhea, vomiting, lack of appetite, constipation, hives, rash, dry skin, occasional abdominal pain, and regurgitation. Two trials^[34,47]^ reported serious adverse events (SAEs). One study^[34]^ noted the intensity of abdominal pain in the placebo group and an otitis media in the symbiotic group as severe. Another trial^[47]^ showed that 1 subject in the active group had SAEs compared with 2 in the control group; the SAEs were all hospitalizations that resolved spontaneously and were believed to be not related to the study product.

## 4 Discussion

In this systematic review, we identified 23 trials involving 6269 children that evaluated the effect of probiotic consumption on the RTIs. All studies were double-blinded, randomized, and placebo-controlled trials with no high risk of bias, as well as no evidence of significant publication bias. Most of trials were conducted in Europe, including France,^[34,35]^ Ukraine,^[36]^ Finland,^[37,38,44,48,52,53]^ Croatia,^[39–42]^ Norway,^[43]^ Sweden,^[50,51,54]^ and Spain.^[46]^ The study products used among these trials included single probiotic strain, such as *L rhamnosus* HN001, *L rhamnosus* GG (ATCC 53103), *B animalis* subsp. *lactis* BB-12, and *L fermentum* CECT5716, or a mixture of several probiotic strains. In addition to prevention studies, 4 treatment trials^[38,50,51,54]^ for AOM with probiotics were also included in this systematic review. Trials with available data of RTIs as outcome were included in the meta-analysis. Pooled data analysis showed that probiotic supplementation significantly decreased the number of subjects with at least 1 RTI episode. We also found that children supplemented with probiotics had fewer numbers of days of RTIs per person, and had fewer numbers of days absent from day care/school compared with children who had taken a placebo. However, there was a statistical heterogeneity among these trials, and subgroup analysis could not elucidate sources of such a statistical heterogeneity (data not shown).

RTIs refer to any of a number of infectious diseases involving the respiratory tract, and are normally further classified as an URTI or a LRTI. Typical URTIs include tonsillitis, pharyngitis, laryngitis, sinusitis, otitis media, certain types of influenza, and the common cold.^[56]^ Symptoms of URTIs can include cough, sore throat, runny nose, nasal congestion, headache, low-grade fever, facial pressure, and sneezing. The most common LRTIs are bronchitis and pneumonia, and are generally more serious than URTIs.^[57]^ RTIs remain a major challenge for global public health by causing morbidity and mortality among children. Global Burden of Disease Pediatrics Collaboration reported that LRTIs were the leading causes of death among younger children aged <5 years in 2013.^[58]^ Children are high-risk group of RTIs who may experience several episodes of RTIs per year, and the duration of symptoms may last for weeks.^[59,60]^ A systematic study showed that there is insufficient evidence for antibiotic use as a means of reducing the risk of both URTI and LRTI.^[61]^ It is very important to decrease the incidence of new episodes of RTIs, shorten the duration time, and reduce symptoms. In this systematic review, we showed that probiotic supplementation could decrease the number of subjects with at least 1 RTI episode and duration of illness.

Potential underlying mechanisms of the action of probiotics on RTIs are not well defined yet. In addition to the local effects of competitively colonizing the gut to exclude potential pathogens, modulating the gut barrier function, and permeability, probiotics have been shown to have various immunomodulatory effects in the host.^[62–64]^ It has been shown that probiotics can influence both innate and adaptive immune responses by producing exopolysaccharides.^[65]^ A study showed that probiotics could increase the leukocyte, neutrophil, and natural killer cell counts and activity.^[66]^ They also have been shown to be able to increase the expression of interleukin (IL)-10 and decrease the inflammatory cytokine expression, such as tumor necrosis factor-α, IL-1β, and IL-8.^[67]^ Furthermore, probiotics can maintain higher salivary immunoglobulin A levels and produce bacteriocins and reuterin, which have antimicrobial activity.^[68]^

In addition to the benefit on the RTIs in children, it has been demonstrated that probiotics can treat or prevent gastrointestinal disorders. Recent meta-analyses showed that *L rhamnosus* GG consumption significantly reduced the duration of diarrhea compared with placebo or no treatment, and decreased the risk of antibiotic-associated diarrhea.^[24,25]^*L reuteri* DSM 17938 administration reduced the risk of necrotizing enterocolitis in preterm infants, as well as reduced crying time in infants with infantile colic in exclusively or predominantly breastfed babies.^[69,70]^ Furthermore, besides the bacterial probiotics, yeast probiotic strains were used to treat gastrointestinal disorders. A study showed that yeast probiotic *S boulardii* administration could significantly reduce the duration of diarrhea, and *S cerevisiae* CNCM I-3856 increased the therapy response rate in patients with IBS.^[23,71]^ The beneficial effects of probiotics have been also reported in urogenital tract infections, allergies, and autoimmune diseases.^[17,21]^

Although several systematic reviews and meta-analyses^[72–78]^ have reported the effect of probiotics in the prevention of RTIs in children in the past few years, the difference with the current meta-analysis should be noted. We included the latest trials published in 2016 that were not included in the previous reports.^[36,53]^ Several reports^[22,75–77]^ did not pool the outcome data for meta-analysis. One meta-analysis reported only the effect of *L rhamnosus* GG for preventing RTIs.^[73]^ And only 1 meta-analysis evaluated the effectiveness of probiotics on the duration of respiratory illness episodes in children pooled with adults, and restricted to the trials using *Lactobacillus* and *Bifidobacterium* strains.^[78]^ In addition, most of reports focused on the prevention effect of probiotics on the RTIs in children; we also included several treatment trials for AOM in the current meta-analysis.^[38,50,51,54]^

There are some limitations in this systematic review. First, the probiotic strain, the duration of regimens, administration forms, doses, and follow-up time differed across the included studies. Second, young children aged <5 years, especially <2 years, are more likely to get RTIs; the trials with the study population age ranging from newborn to 18 years old were included in the systematic review. Most of the trials did not report the outcomes of different age groups; it may cause some statistical bias of the overeffect of probiotics on the incidence of RTIs. Third, we included only trials published in English; other languages, abstracts presented in conferences, and ongoing registered trials were not included. Finally, statistical heterogeneity was present in most of the pooled analyses. Although subgroup analysis was performed, we could not elucidate sources of such a statistical heterogeneity.

## 5 Conclusions

Taken together, the present systematic review and meta-analysis suggested that probiotic consumption may decrease the incidence and illness duration of RTI episode. The optimal probiotic strains, dosing, administration form, time of intervention, and long-time follow-up should be considered in future clinical trials. And studies are needed to explore the mechanisms of such action of probiotics on RTI in children.

## Acknowledgment

The authors thank Dr Juliet C. Peña for her assistance in manuscript revision.

## Supplementary Material

Supplemental Digital Content
