# Supplementary material for: Probiotics for prevention and treatment of respiratory tract infections in children: A systematic review and meta-analysis of randomized controlled trials
Source: Medicine (Baltimore). 2016 Aug 7;95(31):e4509. doi: 10.1097/MD.0000000000004509 (PMC4979858; doi:10.1097/MD.0000000000004509)
Supplement: Supplemental Digital Content [file medi-95-e4509-s001.pdf]

Probiotics for Prevention and Treatment of Respiratory Tract Infections in Children

A Systematic Review and Meta-Analysis of Randomized Controlled Trials

Yizhong Wang, PhD, et al.,

Detailed study search strategy:

Electronic databases MEDLINE/PubMed, Embase, Cochrane Library and Web of Science were searched for records that compared probiotics to placebo in RTIs in children with key words probiotic or probiotics, and respiratory tract infections or respiratory infections, and children or infant. The databases were screened for publications from the earliest available date until April 30, 2016. The detailed search strategies for each database were as follows: MEDLINE/PubMed: (("probiotics"[MeSH Terms] OR "probiotics"[All Fields] OR "probiotic"[All Fields]) AND ("respiratory tract infections"[MeSH Terms] OR ("respiratory"[All Fields] AND "tract"[All Fields] AND "infections"[All Fields]) OR "respiratory tract infections"[All Fields] OR ("respiratory"[All Fields] AND "infection"[All Fields]) OR "respiratory infection"[All Fields])) AND ("child"[MeSH Terms] OR "child"[All Fields] OR "children"[All Fields]). EMbase: 'probiotic'/exp OR probiotic AND respiratory AND ('infection'/exp OR infection) AND ('child'/exp OR child). Web of Science: TOPIC: (probiotics) AND TOPIC: (respiratory tract infections) AND TOPIC: (child). And Cochrane Library: (probiotics) AND (respiratory tract infections) AND child). Finally, 548 records were found, 114 from MEDLINE/PubMed, 215 from

Embase, 75 from Cochrane Library, and 144 from Web of Science. Studies were further selected according to the inclusion criteria in the material and methods part (Figure S1).

Probiotics for Prevention and Treatment of Respiratory Tract Infections in Children

A Systematic Review and Meta-Analysis of Randomized Controlled Trials

Yizhong Wang, PhD, et al.,

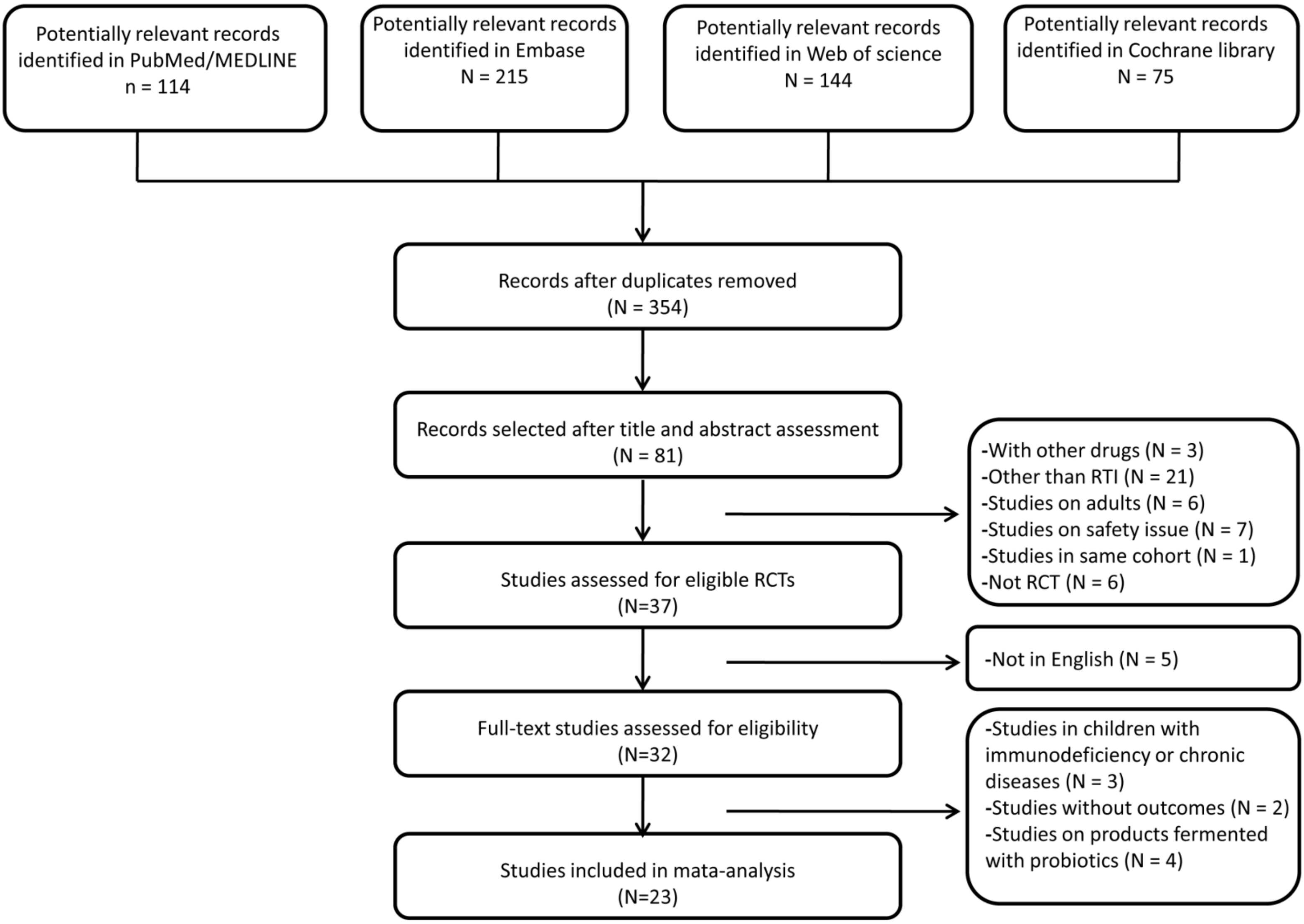

FIGURE S1. Detailed selection process for the studies included in the meta-analysis

Probiotics for Prevention and Treatment of Respiratory Tract Infections in Children

A Systematic Review and Meta-Analysis of Randomized Controlled Trials

Yizhong Wang, PhD, et al.,

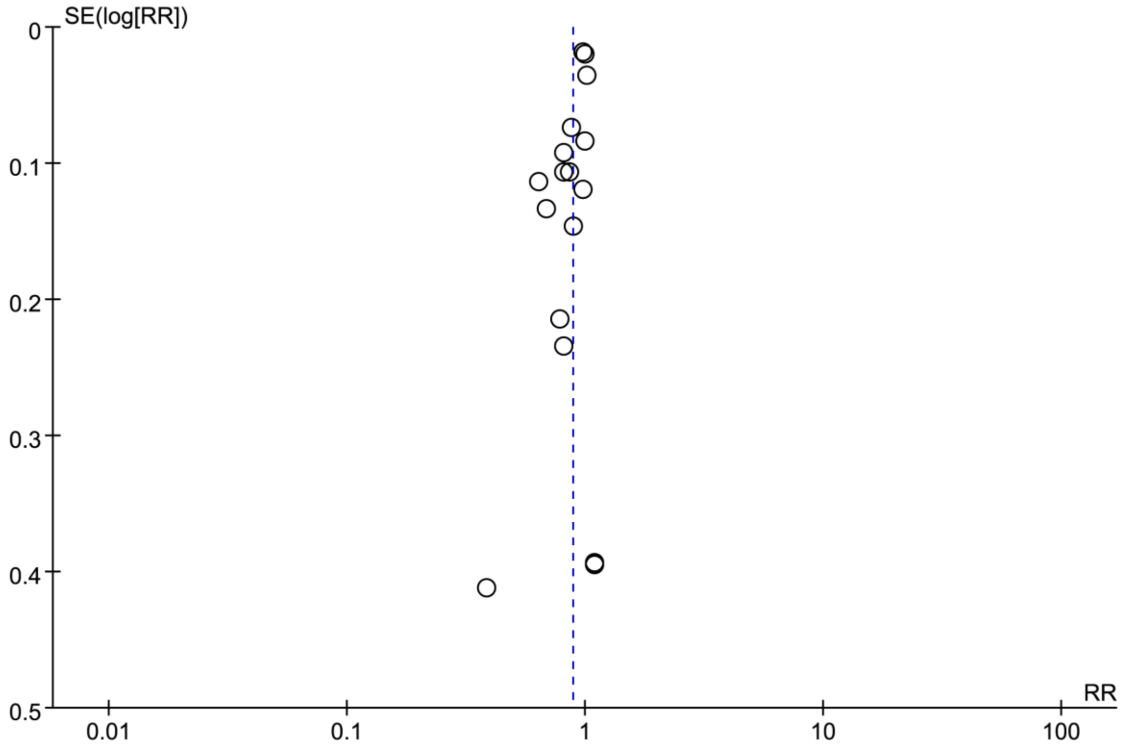

FIGURE S2. Funnel plot of trials of probiotics on the number of subjects had at least one RTI episode

Probiotics for Prevention and Treatment of Respiratory Tract Infections in Children

A Systematic Review and Meta-Analysis of Randomized Controlled Trials

Yizhong Wang, PhD, et al.,

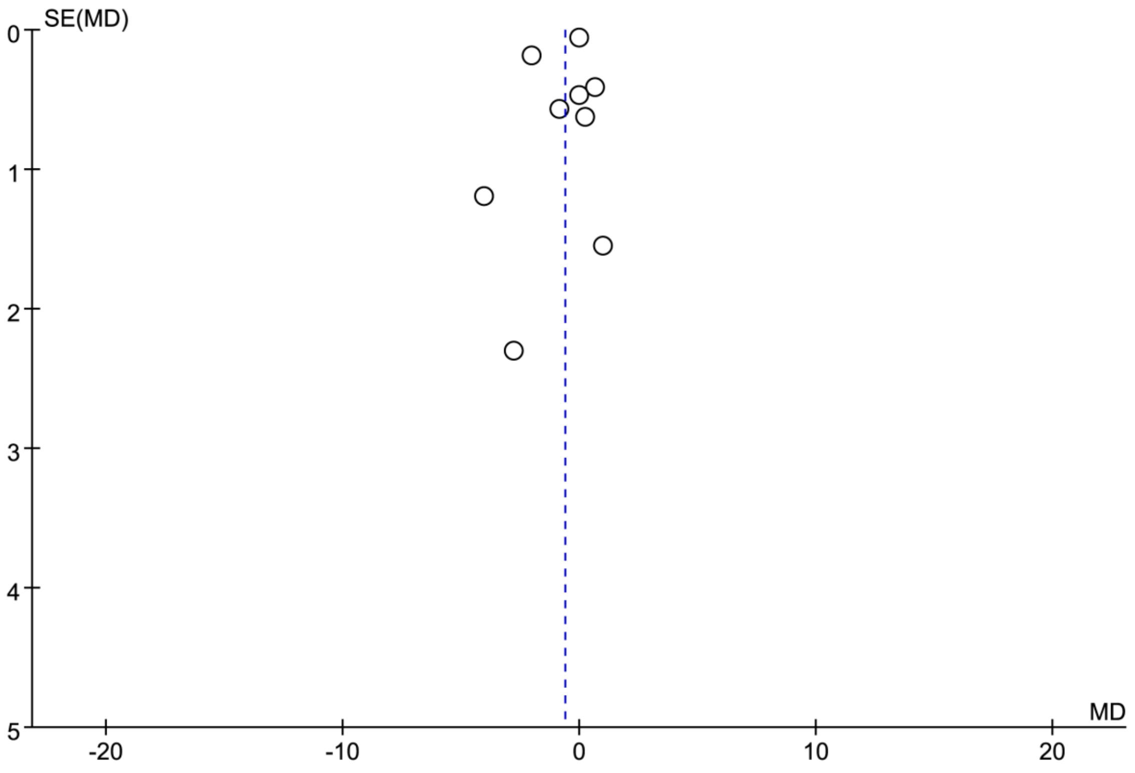

FIGURE S3. Funnel plot of trials of probiotics on the duration of RTI illness episodes

Probiotics for Prevention and Treatment of Respiratory Tract Infections in Children

A Systematic Review and Meta-Analysis of Randomized Controlled Trials

Yizhong Wang, PhD, et al.,

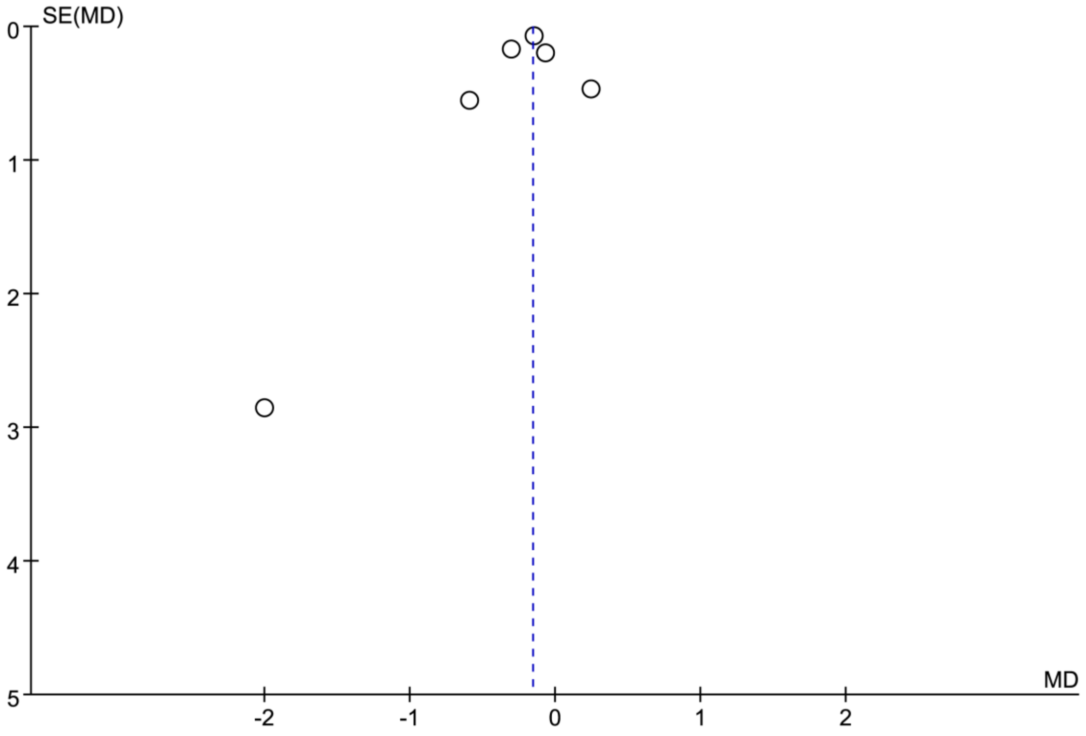

FIGURE S4. Funnel plot of trials of probiotic on the number of days of RTIs illness

Probiotics for Prevention and Treatment of Respiratory Tract Infections in Children

A Systematic Review and Meta-Analysis of Randomized Controlled Trials

Yizhong Wang, PhD, et al.,

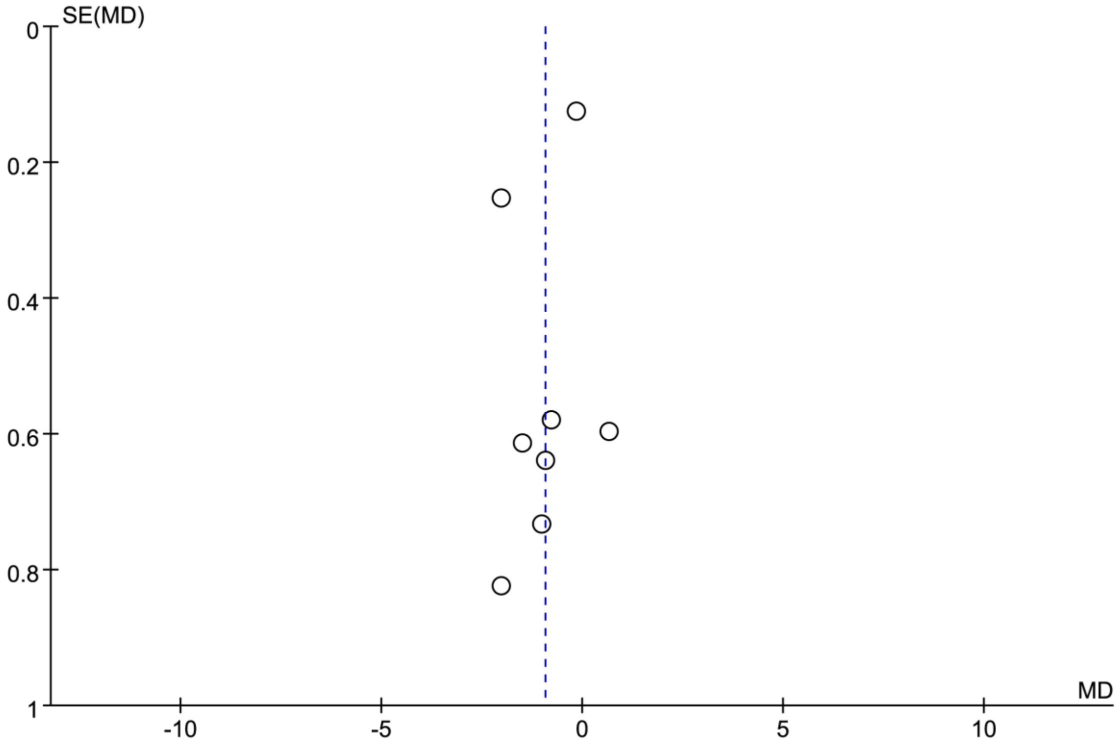

FIGURE S5. Funnel plot of trials of probiotic on the days absent from day care/School

Probiotics for Prevention and Treatment of Respiratory Tract Infections in Children

A Systematic Review and Meta-Analysis of Randomized Controlled Trials

Yizhong Wang, PhD, et al.,

Summary of finding table:

TABLE S1 Summary of finding: probiotics for RTI in children

Probiotics compared with placebo for RTI in children						
Patient or population: Children with RTI; Settings: High- and middle-income countries; Intervention: Probiotics; Comparison: Placebo						
	Illustrative comparative risks* (95% CI)		Relative effect (95% CI)	No of Participants (studies)	Quality of the evidence (GRADE)	Comments
Outcomes	Assumed risk Placebo	Corresponding risk Probiotics				
Number of subjects with at least one illness episode	Study population<br>496 per 1000	441 per 1000 (407 to 476)	RR 0.89 (0.82 to 0.96)	4513 (17 studies)	⊕⊕⊕⊖ moderate[1]	
	Moderate<br>676 per 1000	602 per 1000 (554 to 649)				
Duration of illness episodes	The mean duration of illness episodes ranged across control groups from 0.21 to 20.4 (days)	The mean duration of illness episodes in the intervention groups was 0.6 lower (1.49 lower to 0.3 higher)		2761 (9 studies)	⊕⊕⊕⊖ moderate[1]	
Number of days of illness	The mean number of days of illness ranged across control groups from 0.54 to 21	The mean number of days of illness in the intervention groups was 0.16 lower (0.29 to 0.02 lower)		1672 (6 studies)	⊕⊕⊕⊕ high	
Number of days of absent	The mean number of days of absent ranged across control groups from 0.27 to 7.5	The mean number of days of absent in the intervention groups was 0.94 lower (1.72 to 0.15 lower)		1648 (8 studies)	⊕⊕⊕⊖ moderate[1]	

*The basis for the assumed risk is the median control group risk across studies. The corresponding risk (and its 95% Cl) is based on the assumed risk in the comparison group and the relative effect of the intervention (and its 95% CI).

Abbreviations: CI, Confidence interval; RR, Risk ratio; GRADE, Grading of Recommendations Assessment, Development, and Evaluation.

[1] Because of inconsistency in absolute effects
